# Oil Conductivity, Electric-Field-Induced Interfacial Charge Effects, and Their Influence on the Electro-Optical Response of Electrowetting Display Devices

**DOI:** 10.3390/mi11070702

**Published:** 2020-07-20

**Authors:** Chengdian Jiang, Biao Tang, Bojian Xu, Jan Groenewold, Guofu Zhou

**Affiliations:** 1 Guangdong Provincial Key Laboratory of Optical Information Materials and Technology & Institute of Electronic Paper Displays, South China Academy of Advanced Optoelectronics, South China Normal University, Guangzhou 510006, China; chengdian.jiang@m.scnu.edu.cn (C.J.); j.groenewold@uu.nl (J.G.); guofu.zhou@m.scnu.edu.cn (G.Z.); 2 Shenzhen Guohua Optoelectronics Tech. Co. Ltd., Shenzhen 518110, China; 3 National Center for International Research on Green Optoelectronics, South China Normal University, Guangzhou 510006, China; 4 Van ‘t Hoff Laboratory for Physical and Colloid Chemistry, Debye Institute for Nanomaterials Science, Utrecht University, Padualaan 8, 3584 CH Utrecht, The Netherlands; 5 Academy of Shenzhen Guohua Optoelectronics, Shenzhen 518110, China

**Keywords:** electrowetting display device, oil backflow, charge trapping in fluoropolymer, charge conduction in oil

## Abstract

A pixel in an electrowetting display (EWD) can be viewed as a confined water/oil two-phase microfluidic system that can be manipulated by applying an electric field. The phenomenon of charge trapping in the protective dielectric and conductivity of the oil phase reduce the effective electric field that is required to keep the three-phase contact line (TCL) in place. This probably leads to an oil-backflow effect which deteriorates the electro-optical performance of EWD devices. In order to investigate charge trapping and conduction effects on the device electro-optical response, an EWD device was studied, which was fabricated with a black oil, aiming for a high-contrast ratio and color-filter display. For comparison, we also prepared a device containing a purple oil, which had a lower electrical conductivity. As anticipated, the black-oil device showed faster backflow than the purple-oil device. A simple model was proposed to explain the role of oil conductivity in the backflow effect. In addition, the rebound and reopening effects were also observed after the voltage was switched to zero. The above observations were strongly dependent on polarity. By combining observations of the polarity dependence of the oil conductivity and assuming that negative charges trap more strongly in the dielectric than positive charges, our experimental results on rebound and reopening can be explained. In the AC optical response, the pixel closing speed decreased in time for intermediate frequencies. This is likely related to the phenomenon of charge trapping. It was also found that the periodic driving method could not suppress the backflow effect when the driving frequency was above ~10 kHz. Our findings confirm the significance of the above charge-related effects of EWD devices, which need to be investigated further for better understanding in order to properly design/use materials and driving schemes to suppress them.

## 1. Introduction

Electrowetting displays (EWDs) based on the principle of electrowetting on dielectric (EWOD), have already been demonstrated more than a decade ago [1]. As a reflective display technology, the EWD is expected to bring a comfortable reading experience to the human eye during daylight [2], and to be more energy-saving compared to the liquid-crystal display (LCD) since it does not require backlight when being used under sufficient ambient light. Compared to the electrophoretic display (EPD), the EWD technique has the prospect of video speed because the microfluidic droplet in EWD devices moves fast compared to the pigment-particles in EPD devices.

The EWD technology is still in the research and development phase. Amongst several challenges, the oil backflow phenomenon still hinders the successful commercialization of EWDs. The oil backflow phenomenon refers to the flowing back of the dyed oil in a pixel after switching the pixel on while keeping a constant voltage. This phenomenon reduces the stability of the display [3]. It has been reported that both charge trapping in the dielectric or charge leaking through the dielectric can cause an increase in the contact angle of the EWOD [4]. From the perspective of the electromechanical explanation of electrowetting, both effects reduce the charge accumulation at the liquid/dielectric interface [5,6,7,8], thereby reducing the electrowetting force that keeps the TCL in place. Hence, the backflow phenomenon can result from the charge trapping or leakage processes in the EWD device [9,10,11]. The charge trapping effect has been reported to be related to the charge species, that is, dependent on the driving voltage polarity [12]. The phenomenology of charge trapping is quite rich. For instance, in certain instances it has been observed that charge trapping causes transient strengthening in the electrowetting effect in the EWOD due to the slow relaxation of the trapped charge [7,13]. In addition, certain types of dyes and an increase in concentration can strengthen the oil backflow [3,14]. Taking the electromechanical standpoint into account, charge leaking through the oil and subsequent accumulation on the oil/dielectric interface screen the effective electric field around the TCL [8], which is the most likely reason behind the backflow. Moreover, protonation of dyes with amino groups [15,16,17,18,19,20,21] and formation of inverse micelles in the oil [22,23] could also lead to significant charge conduction in the oil. As a result, both mechanisms that may occur in the dyed oil could facilitate the backflow.

In this work, the device structure was the same as that in the previous report [24]. We defined the open ratio (*OR*) as the ratio of the white area to the total area of a pixel. The responses of the open ratio to constant voltage (DC) were measured. Y. Y. Guo and coworkers have discussed the influence of the oil conductivity on the oil-backflow effect by varying the purple oil’s concentration [14]. In this work, we further investigated the electro-optical response of devices based on different colored oil. Hence, the oil backflow effect and its dependence on the oil and the voltage polarity was characterized. In addition to the backflow effect, a rebound of the open ratio and the reopening of the device were observed. It was also found that the initial opening was dependent on the voltage polarity. Driving the devices using a periodic square-wave voltage pulse (AC) was performed. The backflow could be suppressed in a proper frequency range. Furthermore, a stable AC optical response state switching between the maximum and minimum open ratio was observed. To reach this state, several cycles were required. This transition stage is likely related to the phenomenon of charge trapping. Phenomenological explanations of the above experimental results based on the charge trapping and charge conduction effects being proposed aim to be consistent with the observations on the black and purple oil devices.

## 2. Experimental

### 2.1. EWD Device Fabrication

The fabrication steps of the EWD are shown in Figure S1. First, the ITO/glass substrate (ITO sheet resistance ~100 Ω/□) was cleaned in the standard cleaning line of liquid crystal display manufacture (Figure S1a). A layer of amorphous fluoropolymer (FP) (AFX 1600, Chemours, *ε_r_* = 1.934) was spin-coated on the substrate with the thickness ~0.8–0.9 µm (Figure S1b). The FP surface was activated using reactive ion etching (RIE) to change the hydrophobic state to the hydrophilic state, thereby promoting the adhesion of a layer of n-type photoresist (PR) on the FP (Figure S1c). The PR was then spin-coated on the FP at 1500 rpm. After the photolithography process, the pixel walls were formed (width: ~15 µm, height: ~5.5 µm), as shown in Figure S1d. Next, the substrate was heated above the glass transition temperature of the FP (160 °C) to reverse the FP surface back to the hydrophobic state (Figure S1e).

The colored oil was then uniformly filled into the pixels through a raster filling method under water at low speed (~1 mm/s), as shown in Figure S1f. Two kinds of oil were used in the devices, a purple oil and a black oil. The purple oil was formulated by dissolving a non-polar purple dye [25] in decane (*C*_10_*H*_22_, *ε_r_* = 2.2) at the concentration of 0.21 M. The black oil consisted of three different dyes (magenta [21], cyan, and yellow [26]) dissolved in decane at the concentrations of 6.1 wt%, 5 wt%, and 5 wt%, respectively. All of these dyes have larger molecular polarities than the purple dye. All of the dyes were synthesized in our laboratory. Biao Tang and coworkers showed the synthesis process of the cyan and yellow dyes [27]. The synthesis process of the purple and magenta dyes is shown in SI. The molecular structure of the dyes is shown in Figures S2–S5. Subsequently, the cells were filled with water and covered with a top ITO/glass plate. Finally, the device was edge-sealed using a pressure sensitive adhesive (PSA), gluing the bottom and top plates together (Figure S1g). The device and microscope images with different oils are shown in Figures S6 and S7, respectively.

### 2.2. EWOD Sample (for Contact Angle Measurement) Fabrication

The fabrication steps of the EWOD samples are shown in Figure S8. First, the ITO/glass substrate (ITO sheet resistance: ~100 Ω/□) was cleaned in the standard cleaning line (Figure S8a). A layer of amorphous fluoropolymer (FP) was spin-coated (1000 RPM for 60 s) on the cleaned ITO/glass substrate, and a layer with the thickness of ~1 µm was obtained (Figure S8b). This layer was pre-dried for 3 min on a hot plate at 85 °C, then was annealed in an oven at 185 °C for 30 min (Figure S8c). After that, the surface of the insulating layer was sealed with four pieces of ITO glass to form a container with a volume of 3 cm × 3 cm × 3 cm (Figure S8d).

Water was filled into the container with a height of ~1 cm, and then injected with 5 μL of a yellow oil on the surface of the insulating layer (Figure S8e). The yellow oil was formulated by dissolving a yellow dye [26] in decane.

### 2.3. Oil Conductivity Measurement

Guangzhou Zihui DPCM-11/YX1154 (accuracy: 0.01 pS/m) was used to measure the oil conductivity. Then, 100 mL of formulated oil was filled into the testing cell. The conductivity of the oil was measured with 5 V DC within 3 sec. The value was the average of two readings with different electrode polarities. All the measurements were conducted in a clean room at a temperature of 25 ± 2 °C and relative humidity of 50%.

### 2.4. Electrical and Optical Measurements

A high-speed camera (MIRO M110) with a sampling rate of 14,000 frame/sec was focused on the measured EWD devices for recording a video of it. The EWD devices were driven by a constant voltage (DC) generated by a voltage source (Tektronix 2000) and a voltage amplifier (A400, FLC Electronics AB). The positions of the devices and the camera were fixed to ensure that the same area was recorded. The voltage was applied to the top ITO plate, and the bottom one was grounded. The current was measured using a pico-ammeter (Keithley 6487) controlled by a LabVIEW program. The current sampling speed was 50 data points/sec. For the purple-oil device, a 25 V driving voltage was adopted to ensure that the pixels were open. When the voltage was larger than 25 V, oil flowed over the pixel wall. Hence, measurements at a larger voltage were not conducted. For the black-oil device, 30 V was required to open the pixels, and the voltage at which the oil flowed over the pixel wall was 50 V. Hence, both 30 V and 44 V, and both positive and negative polarities, were employed to drive the device. The voltage was set as 0 V first, then jumped to the driving voltage and maintained it for a certain amount of time, and was set back to zero at the end. The duration of the driving voltage was controlled by looking at a timer and manually switching on/off the voltage source. After every time the device was tested, the top and bottom ITO plates were both connected to the ground to release the excess charge. The voltage-dependence of capacitance of the devices was measured using an LCR meter (TH2828) before and after the above measurements to characterize the degree of the device degradation and the reliability of the measurement results.

### 2.5. Optical Measurement Data Processing

For data processing purposes, an image recognition and processing program written in C++ was adopted to derive the open ratio (*OR*) of a device pixel in each frame. By setting a threshold gray value to distinguish whether an image pixel in the frame belonged to the water or the oil, the program counted the total image-pixel number representing the water area (*N_water_*) and the total image-pixel number representing the oil (*N_oil_*). The total number of image pixels of one device pixel ($N_{water}+N_{oil}$) is 166 × 166. Then, *OR* was obtained by $OR=100\%\cdot N_{water}/27556$.

### 2.6. Contact Angle Measurements

A voltage was applied between the water in the container and the ITO substrate, and varied from 0 V to 100 V with a voltage step of 2 V (voltage holding time: 1 s) to manipulate the contact angle of water to the FP. In order to prevent the insulator from being broken down, the maximum voltage was set to 100 V [28]. The voltage was generated by a pico-ammeter (Keithley 6487). Contact angles were monitored and recorded by a contact angle goniometer (OCA 15) while varying the voltage.

### 2.7. Oil–Water Surface Tension Measurements

The pendant drop method was used to measure the oil–water surface tension. A syringe was inserted into a 5 cm × 5 cm × 5 cm container filled with water, and the oil was injected into the water by a syringe pump. The oil drop was suspended in the water due to the oil density being smaller than water. A camera equipped with Dataphysics OCA Pro 15 was employed to take a picture when the oil drop was about to leave the tip of the syringe. All the measurements were conducted in a clean room at a temperature of 25 ± 2 °C and relative humidity of 50%.

## 3. Results and Discussion

### 3.1. Effect of Driving Polarity and Oil Conductivity on Optical Response

#### 3.1.1. Oil Conductivity Effect on EWD Devices’ Behavior

**Figure 1:**
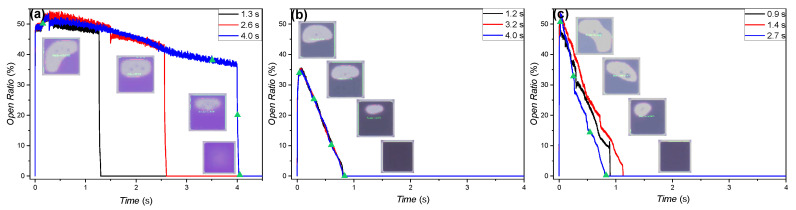
Open ratio measurement results of the purple- and black-oil devices. (**a**) For the purple-oil device, the applied voltage was 25 V. The voltage was maintained for a holding time period of 1.3 s (black), 2.6 s (red), and 4.0 s (blue). (**b**) For the black-oil device, the applied voltage was 30 V. The holding time was 1.2 s (black), 3.2 s (red), and 4.0 s (blue). (**c**) For the black-oil device, the applied voltage was 44 V. The holding time was 0.9 s (black), 1.4 s (red), and 2.7 s (blue).

Figure 1a shows the experimental result of the purple-oil device measured at 25 V, and Figure 1b,c show the experimental results of the black-oil device measured at 30 V and 44 V, respectively. The holding time was manually set to different values. After the holding time, the voltage was switched off. While 25 V was applied, a backflow with a slope of ~3%/sec is shown in Figure 1a. The speed of the backflows shown in Figure 1b,c are both much faster than that shown in Figure 1a. In addition, the initial *OR* of Figure 1b is much smaller than that of Figure 1a,c. There is no notable difference among the results of different holding times. In summary, the black-oil device shows a much faster oil backflow than the purple-oil device. In addition, in order to achieve the same initial *OR*, the black-oil device needed a larger driving voltage.

According to the investigation reported in [29], screening the electric field across the oil film due to the movement and distribution of the charge in the oil also severely affects the rupture process of oil film. The measured oil conductivity was 2.3 × 10^−^^12^ S/m for the newly prepared purple oil, and 1.7 × 10^−10^ S/m for the newly prepared black oil. Considering that the black oil had larger conductivity than the purple oil, we speculate that the initial *OR* of the black-oil device was also suppressed by the charge conduction in the oil film upon applying the voltage.

From an electromechanical point of view, the electrowetting force is decreased due to a decrease of the accumulated charge density at the water/FP interface [6]. This decrease can be a result of the charge getting trapped in the dielectric. Another mechanism that reduces the magnitude of the dielectric force is that charges accumulate on the oil/FP interface as well. If this happens, the electrowetting force keeping the TCL in place reduces, and backflow will eventually occur [14]. The accumulated charge density on the oil/FP is expected to be primarily caused by charge conduction through the oil phase, which implies that the conductive properties of the oil should play a critical role in the fast oil backflow effect. So, the faster oil backflow of the black-oil device is highly likely to be related to the role of the charge transport in the oil.

**Figure 2:**
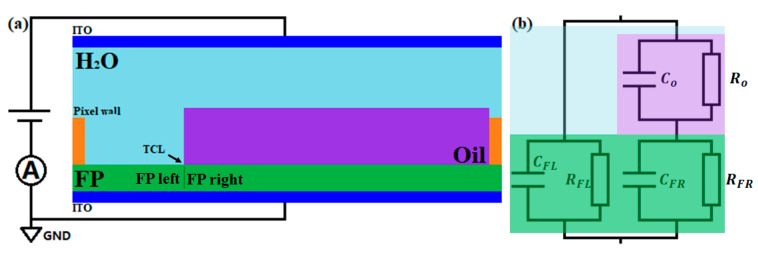
Schematic of the electrowetting display (EWD) device when a driving voltage is maintained (**a**) and the equivalent circuit (**b**). In (**a**), the contracted oil part is simplified to a column. The fluoropolymer layer is divided into the left and right part by the three-phase contact line (TCL). In (**b**), the colored regions represent different parts of the device in (**a**) correspondingly.

According to the energy minimization method, the electrowetting effect on dielectric is driven by the energy resulting from the charge redistribution from the voltage source to the droplet on dielectric [6]. Concerning the electrowetting effect in the EWD device, when the charge leaks from the water to the oil, the driving energy is therefore decreased, degrading the driving performance. The electrowetting driving energy is measured by the difference of the stored capacitive energy per unit area of the water/FP/ITO and of the oil/FP/ITO. Hence, in the following, the difference of the two energies, that is, the energy difference between both sides of the three-phase contact line (TCL), was calculated based on the equivalent circuit model of the device shown in Figure 2, and the effect of the oil conductivity on the electrowetting performance is demonstrated. To simplify the energy calculation regarding the oil, the shape of the oil was assumed to be a column instead of a spherical geometry, as shown in Figure 2a, and the water was assumed to be a perfect conductor.

The energy difference Δ*E* is defined as $-E_{water}+E_{oil}$. It can be interpreted as the force on the contact line in the direction of increasing the water/FP area where $E_{water}=-\frac{1}{2}C_{FL}V_{total}{}^{2}$ and $E_{oil}=-\frac{1}{2}C_{o}V_{o}{}^{2}-\frac{1}{2}C_{FR}V_{FR}{}^{2}$. *V_total_* is the voltage applied across the device, and *V_o_* and *V_FR_* are the voltages distributed across the oil and the right part of the FP, respectively. Hence, $V_{total}=V_{o}+V_{FR}$, $V_{o}=\frac{R_{o}}{R_{o}+R_{FR}}V_{total,}$ and $V_{FR}=\frac{R_{FR}}{R_{o}+R_{FR}}V_{total}$. The circuit parameters are $C_{o}=\varepsilon_{0}\varepsilon_{oil}\frac{S_{R}}{d_{oil}}$, $R_{o}=\frac{1}{\sigma_{oil}}\frac{d_{oil}}{S_{R}}$, $C_{FR}=\varepsilon_{0}\varepsilon_{FP}\frac{S_{R}}{d_{FP}}$, $R_{FR}=\frac{1}{\sigma_{FP}}\frac{d_{FP}}{S_{R}},\;C_{FL}=\varepsilon_{0}\varepsilon_{FP}\frac{S_{L}}{d_{FP}}$, and $R_{FL}=\frac{1}{\sigma_{FP}}\frac{d_{FP}}{S_{L}}$, where *ε*_0_ is the vacuum permittivity, *ε_oil_* and *ε_FP_* are the relative permittivities of the oil and FP, respectively, *σ_oil_* and *σ_FP_* are the electrical conductivities of the oil and FP, respectively, and *d_oil_* and *d_FP_* are the thicknesses of the oil and FP, respectively. *S_L_* and *S_R_* are the areas of the left and right, corresponding to the areas of the water/FP and oil/FP interfaces, respectively. Because the FP thickness (0.8–0.9 μm) is much smaller than the pixel length (~150 μm), the energy due to the fringe field at the edge of the water–FP–ITO and oil–FP–ITO capacitors is neglected in this report [6]. Therefore, the capacitive energies can be replaced by corresponding area-normalized energies of *E_water_* and *E_oil_*, and the difference in the energy per unit area between the water and oil, Δ*E_S_*, defined as $\frac{-E_{water}}{S_{L}}+\frac{E_{oil}}{S_{R}}$ and expressed in Equation (1), is interpreted as the driving force. In this report, we neglect the change in *σ_oil_* and *σ_FP_* due to the water. To plot Δ*E_S_* as a function of *σ_oil_*, the parameters were set as follows [29]: *d_oil_* = 10 µm, *d_FP_* = 0.85 µm, *ε*_0_ = 8.85 × 10^−12^ F/m, *ε_oil_* = 2.2, *ε_FP_* = 1.934, and *V_total_* = 30 V. The plot is performed at three *σ_FP_*, 3 × 10^−11^ S/m, 3 × 10^−12^ S/m, and 3 × 10^−13^ S/m.

(1) $$\Delta E_{S}=\frac{\varepsilon_{0}\varepsilon_{FP}V_{total}{}^{2}}{2d_{FP}}\left[\frac{\frac{\sigma_{FP}}{\sigma_{oil}}\frac{d_{oil}}{d_{FP}}\left(2+\frac{\sigma_{FP}}{\sigma_{oil}}\frac{d_{oil}}{d_{FP}}\right)}{{\left(1+\frac{\sigma_{FP}}{\sigma_{oil}}\frac{d_{oil}}{d_{FP}}\right)}^{2}}-\frac{\frac{d_{FP}}{d_{oil}}\frac{\varepsilon_{oil}}{\varepsilon_{FP}}}{{\left(1+\frac{\sigma_{oil}}{\sigma_{FP}}\frac{d_{FP}}{d_{oil}}\right)}^{2}}\right]$$

**Figure 3:**
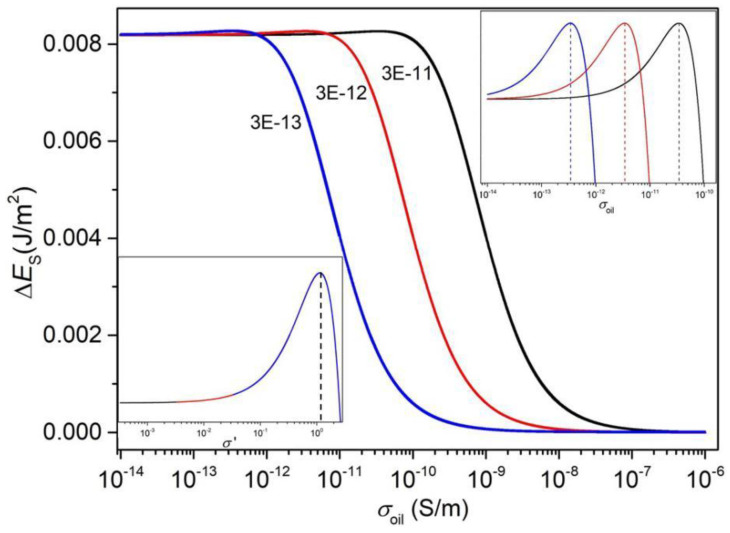
Effect of the oil conductivity on the energy difference per unit area. The plot is based on Equation (1) at three *σ_FP_*, 3 × 10^−11^ S/m (black), 3 × 10^−12^ S/m (red), and 3 × 10^−13^ S/m (blue), which is also denoted by the numbers beside the curves. The top right is the zoom-in of the local maximum of Δ*E_S_*. The dashed lines denote the position of the peaks. The bottom left is also the zoom-in, but the horizontal axis is normalized to *σ_FP_* ($\sigma^{\prime }\equiv \sigma_{oil}/\sigma_{FP}$).

As shown in Figure 3, the energy difference firstly increases slightly with the oil conductivity. After reaching a maximum at certain oil conductivity, there follows a dramatic decreasing trend as *σ_oil_* increases further, until reaching zero eventually. The transition conductivity increases with the FP conductivity. From the two insets of the figure, we can see that the transition *σ_oil_* is a little bit larger than *σ_FP_*, and the local maximum always appears at the same normalized oil conductivity *σ′* ($\sigma^{\prime }\equiv \sigma_{oil}/\sigma_{FP}$). The result shows that increasing the oil conductivity deteriorates the driving energy. Based on the result, *σ_FP_* can be a criterion to judge whether *σ_oil_* is too large to realize a sufficient electrowetting phenomenon in the device. The oil should be prepared to be less conducting than the FP to ensure the driving performance, and is desired to be not too much more electrically resistive than the dielectric layer, for achieving the best performance. In our devices, the FP conductivity was on the order of 10^−13^ S/m [29], while the measured oil conductivity was 2.3 × 10^−^^12^ S/m for the newly prepared purple oil and 1.7 × 10^−10^ S/m for the newly prepared black oil. Hence, we believe that the increase in the black-oil conductivity plays a significant role in the faster oil backflow of the black-oil device compared to the purple-oil device.

#### 3.1.2. Charge Trapping Effect on EWD Devices’ Behavior

**Figure 4:**
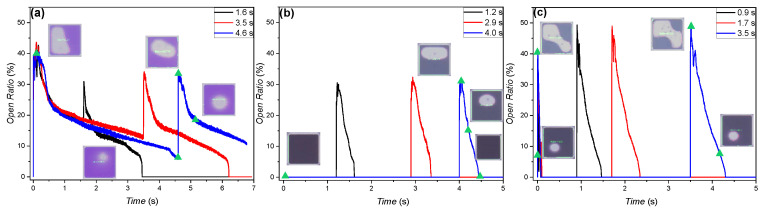
Open ratio measurement results of the purple- and black-oil devices. (**a**) For the purple-oil device, the applied voltage was −25 V. The voltage was maintained for a holding time period of 1.6 s (black), 3.5 s (red), and 4.6 s (blue). (**b**) For the black-oil device, the applied voltage was −30 V. The holding time was 1.2 s (black), 2.9 s (red), and 4.0 s (blue). (**c**) For the black-oil device, the applied voltage was −44 V. The holding time was 0.9 s (black), 1.7 s (red), and 3.5 s (blue).

Figure 4 shows the experimental results of the purple-oil and black-oil devices measured at voltages with opposite polarity to those related to Figure 1. The backflow shown in Figure 4a,c is faster than the result in Figure 1a,c, and the initial *OR* of Figure 4a,c is much smaller than that of Figure 1a,c; the black-oil device is not even open under −30 V, as shown in Figure 4b. In addition, *OR* rebounds for the purple-oil device, as shown in Figure 4a. It is also noted here that the pixel opens for the black-oil device, as shown in Figure 4b,c, at the moment the negative driven voltage is set back to zero. In this work, the phenomenon that the black-oil device opens as soon as the negative voltages are switched to zero is hereinafter called the reopening phenomenon.

The smaller initial *OR* can be explained by a built-in negative voltage due to the adsorption to the FP of the negative charge in the water during the under-water filling process of the oil, which was reported in [12]. Considering the electromechanical point of view, the decrease of the charge density at the water/FP interface can also be due to the accumulated charge being gradually trapped into the FP or a leak through the dielectric layer, thereby causing the backflow effect. Taking account of the porous property of the FP, the charge trapping effect is likely to occur in our EWD devices. It has been reported that the rebound is a strong indication of significant charge trapping in the FP [30]. The provided explanation is as follows: when the voltage is set to zero, the trapped charge in the FP introduces counter ions at the FP/water interface, generating a significant electrowetting force. Similarly, the significant charge trapped at the oil/FP interface can also cause the reopening effect. The above result suggests that the negative charge is trapped in the FP more significantly than the positive charge [31].

#### 3.1.3. Effect of the Oil Conductivity on the Wettability of Conductive Liquid

**Figure 5:**
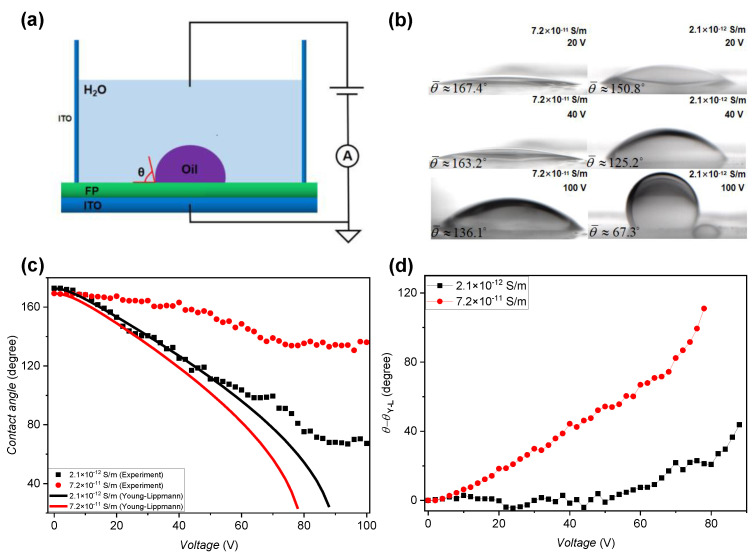
Schematic of the electrowetting on dielectric (EWOD) sample, the contact angle measurement setup, and the contact angle measurement results. (**a**) EWOD sample and the contact angle measurement setup. (**b**) Photos of the oil droplet, taken when V = 20 V, 40 V, and 100 V and at two oil conductivities. (**c**) The measured (symbol) and calculated (line) voltage dependence of the contact angle. The oil conductivity is 2.1 × 10^−12^ S/m (black) and 7.2 × 10^−11^ S/m (red). (**d**) The voltage dependence of the difference between the experimental and theoretical values for the two oil conductivities.

It has been shown in Figure 3 that the driving energy at the TCL decreases as oil conductivity increases, which means that enhancing the oil conductivity may deteriorate the wettability of the conductive liquid on the fluoropolymer. We conducted a contact-angle measurement experiment to show the effect of oil conductivity on the wettability of water. The measurement set-up is shown in Figure 5a. The contact angle of water to the FP, as indicated by *θ* in Figure 5a, was manipulated by applying a voltage between the water and ITO substrate. In this experiment, the voltage was varied from 0 V to 100 V with a voltage step of 2 V (voltage holding time: 1 s). We prepared oil with two concentrations (5 wt% and 20 wt%) to have different oil conductivities. The measured surface tension was 34.79 mN/m and 27.35 mN/m, and the measured oil conductivity was 2.1 × 10^−12^ S/m and 7.2 × 10^−11^ S/m, respectively.

Figure 5b,c show the contact angle measurement results at different oil conductivities and voltages. As the voltage increases from 0 V to 100 V, the contact angle decreases, and the oil droplet was more and more squeezed. The contact angle of the larger oil conductivity is always larger than that of the smaller oil conductivity at the same voltage. The theoretical contact angle, which is plotted in Figure 5c in lines, was calculated based on the Young–Lippman equation [6]:

(2) $$cos\theta =cos\theta_{Y}+\frac{\varepsilon_{0}\varepsilon_{r}}{2d\gamma_{ow}}V^{2}\;\;$$

where *V* is the voltage, *θ* is the contact angle of water to the FP when *V* is applied, *θ_Y_* is the static contact angle when V=0, *ε_r_* and *d* are the relative dielectric constant and thickness of the FP, and *γ_OW_* is the oil–water surface tension since the oil phase replaces the vapor phase. For the results of *σ*_oil_ = 2.1 × 10^−12^ S/m, the calculated value fits the measured result well until the voltage is ~50 V. When the voltage is larger than 50 V, the measured value is larger than the theoretical value and the deviation increases with the voltage. For the results of *σ*_oil_ = 7.2 × 10^−11^ S/m, the measured contact angle deviates from the theoretical value starting from 6 V, as shown in Figure 5c,d. In summary, the contact angle measurement result shows that the measure contact angle is larger than the theoretical value, indicating that the electrowetting effect is suppressed. The deviation becomes larger as the voltage increases. The result also shows that the deviation is larger for the higher oil conductivity.

In the above discussion, we ascribe the suppressing in the electrowetting mainly to the charge trapping effect in the FP and the charge conducting in the oil. Since we used the same materials and processing method for the FP layer and conductive liquid, we neglect here the difference in the influence of the charge trapping effect on the electrowetting effect in the experiments of both oil conductivities. Based on this, we can conclude that the electrowetting effect is stronger when the oil conductivity is smaller.

#### 3.1.4. A Phenomenological Model

**Figure 6:**
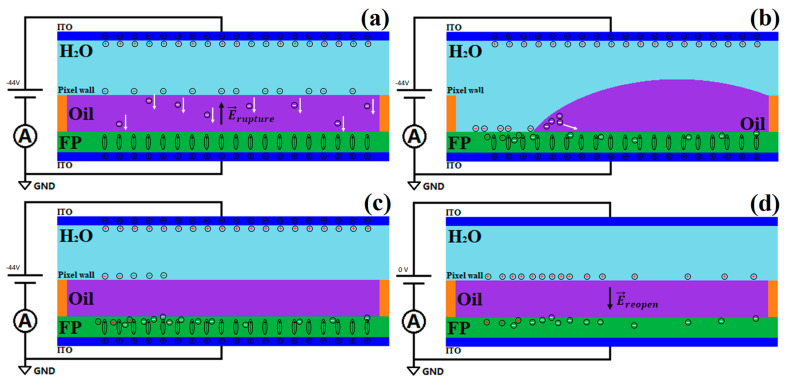
Schematic of a phenomenological model to explain the open ratio responses of −44 V. In each subfigure, the bottom ITO plate (dark blue) is always grounded. (**a**) −44 V is applied to the top ITO plate. Negative (black ⊖) and positive (black ⊕) charges are accumulated on the top and bottom ITO plates, respectively, aligning the dipoles (arrow in ellipse) in the FP (green). Negative (red ⊖) and positive charges (red ⊕) in the water (light blue) move to the water–oil (purple) interface and the top ITO plate, respectively. Some negative charges (white ⊖) flow through the oil, indicated by the white arrows, and then are trapped in the FP, decreasing the electric field, which ruptures the oil film and the electrowetting force as well. (**b**) After the opening of a pixel, more charges are trapped in the FP (red ⊖) or transported in the oil (white ⊖), leading to significant oil backflow. (**c**) The pixel is closed due to the oil backflow. The trapped negative charges mostly remain in the FP. (**d**) The charges on the ITO plates are discharged and the dipoles in the FP are relaxed when the voltage is set to zero. Nevertheless, the positive charges (red ⊕) in the water accumulate at the water–oil interface due to the attraction of the trapped charges, which do not discharge as soon as the voltage is set to zero, thereby generating an electric field which ruptures the oil film again, reopening the pixel.

Based on the above discussion, a phenomenological model is proposed to qualitatively explain how the performance of the black-oil EWD devices is affected by the charge trapping in the FP and the charge transport in the oil. Figure 6 is a cartoon of the model. When a negative voltage is applied to the top ITO plate, the negative and positive charges accumulate on the top and bottom ITO plates, respectively, causing the polarization of the FP. In addition, the negative and positive charges in the water move to the water–oil interface and the top ITO plate, respectively. As demonstrated in Figure 6a, some of the negative charges are transported through the oil due the electrical conductivity of the oil, and then are trapped in the FP, weakening the electric field across the oil, and thereby impeding or even preventing the rupture of the oil film [29]. This can explain why the pixels opened slightly when −44 V was applied and did not open when −30 V was applied. The reason for the larger initial *OR* when +30 V and +44 V were applied then signifies that the oil film was probably less conducting to the positive charge. It could also be mainly due to the build-in voltage mentioned above. It cannot be determined here whether the polarity-dependent conductivity or the built-in voltage plays a more significant role regarding the polarity-dependent initial *OR*. When the pixels were open, the model proposed by Y. Y. Guo et al. indicated that the charge conduction in the oil was able to decrease the electrowetting force [14], which neglected the charge trapping in the FP. Hence, both factors could lead to severe oil backflow, although the voltage remained applied, as shown in Figure 6b,c. When the voltage was set to zero, the trapped charges did not relax immediately. The positive charges in the water were therefore attracted to the water–oil interface, shown in Figure 6d, forming an electric field which ruptured the oil film again and resulted in the reopening phenomenon. The reason that there was no reopening phenomenon implies that the trapped positive charges in the FP were more easily relaxed [31].

### 3.2. Driving Scheme Influence on the Interfacial Dynamics and Charge Trapping

**Figure 7:**
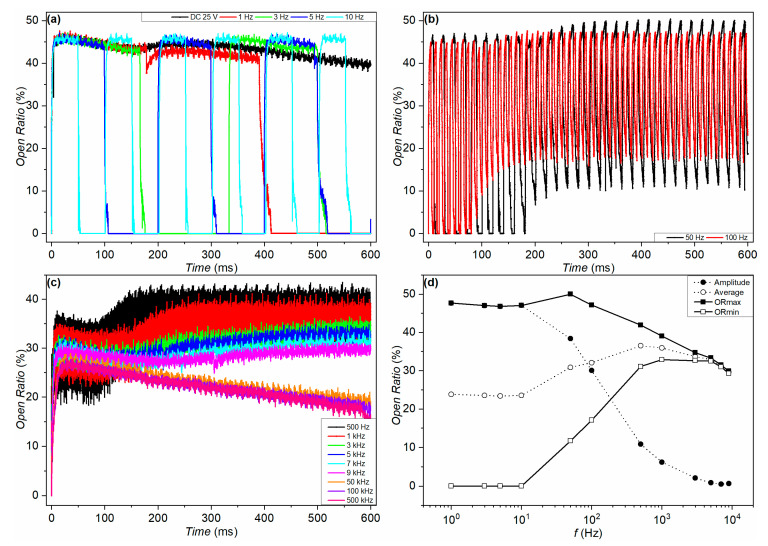
Open ratio response of the purple-oil device. The device was driven by a square-wave voltage (0 V −> 25 V −> 0 V) of different frequencies. (**a**) Time dependence of the open ratio, measured at DC (black), 1 Hz (red), 3 Hz (green), 5 Hz (blue), and 10 Hz (cyan). (**b**) Time dependence of the open ratio, measured at 50 Hz (black) and 100 Hz (red). (**c**) Time dependence of the open ratio, measured at 500 Hz (black), 1 kHz (red), 3 kHz (green), 5 kHz (blue), 7 kHz (cyan), 9 kHz (magenta), 50 kHz (orange), 100 kHz (violet), and 500 kHz (pink). (**d**) Frequency dependence of the amplitude, average, maximum, and minimum of the open ratio.

It is our assumption that the charge trapping and protonation effects are reversible by resetting the voltage. We therefore used a periodic square-wave voltage pulse (AC) to drive the devices in order to suppress the backflow effect. Figure 7 shows the open ratio of the purple-oil device. The device was driven at different driving frequencies. The maximum and minimum open ratio (denoted as *OR*_max_ and *OR*_min_, respectively) are also calculated by averaging the maximum and minimum *OR* of all the periods for each frequency. The amplitude of the open ratio (denoted using *OR*_am_) is defined as the difference between *OR*_max_ and *OR*_min_. The average of the open ratio (denoted using *OR*_av_) is derived by averaging *OR*_max_ and *OR*_min_. Concerning the data in Figure 7a, because of the oil backflow, only the initial *OR* of each period was taken as *OR*_max_. *OR*_min_ was zero. For the data in Figure 7b,c, only the latter stable regime was considered when calculating *OR*_max_ and *OR*_min_. The frequency dependence of *OR*_max_, *OR*_min_, *OR*_am,_ and *OR*_av_ are plotted up to 9 kHz, which is shown in Figure 7d. The faster rising of *OR*_min_ and slower decreasing of *OR*_max_ between 50 Hz and 500 Hz causes an increase in *OR*_av_. Then, the decrease of both *OR*_max_ and *OR*_min_ lead to a decrease in *OR*_av_ with the frequency after 500 Hz. *OR*_am_ keeps decreasing, with the frequency starting from 10 Hz, due to the approaching of *OR*_min_ to *OR*_max_.

When the frequency was low (DC ~10 Hz), the rise and descent of the open ratio followed the voltage switching. There was a gradual decrease of the open ratio when the voltage was on at low frequencies (see Figure 7a). It is worth mentioning that the open ratio always rose to ~46% at the beginning of each period in the results of 3 Hz, 5 Hz, and 10 Hz. This suggests that the deterioration due to the oil backflow is reversible by resetting the voltage.

For the data of 50 Hz shown in Figure 7b, there is a transition regime bridging the initial and the final stable switching state. At the initial stage, the switching of the open ratio still follows the periodic change of the voltage, rising up to ~46% and dropping down to zero. As the number of the driving cycles increases, the dynamic behavior enters a transition regime. *OR*_min_ increases as the cycle number increases. *OR*_max_ also increases with the driving cycle in this regime, but less significantly than *OR*_min_ does. The transition regime gradually evolves to a stable switching state with a smaller *OR*_am_.

During the transition stage, the increase in *OR*_min_ indicates that the closing speed of the device becomes slower in time. It has been mentioned in the literature that charge trapping can cause the slow closing phenomenon of the EWD device, similar to how charge trapping leads to the rebound effect [9,11,30,32,33]. The transition regime therefore implies an asymmetric degree of charge trapping and detrapping, causing a continuous increase of the number of the trapped charges in the dielectric, while the device is being driven periodically.

The critical flicker fusion rate is about 50–90 Hz [34]. Mainstream research recognizes that flickering at a frequency larger than the critical flicker fusion rate is a visually stable state; when the frequency is larger than this, humans perceive a stable image without flicker artifacts. As discussed, oil backflow can be reversed by resetting the voltage. However, while using the resetting, the initial open ratio became less than that shown in Figure 7a,b. One reason is that the time period where the driving voltage is “on” is less than the response time (about 10–20 ms) of the EWD device. As a result, at higher frequencies, the oil film has insufficient time to fully open during the time that the voltage is switched on. So, as the frequency increases further after 100 Hz, the initial open ratio decreases further, shown in Figure 7c,d. The short periods also cause the open ratio to be not fully closed, resulting in an *OR* that just oscillates around a certain value with a relatively small *OR*_am_. As shown in Figure 7c,d, the larger the frequency, the smaller the *OR*_am_. This leads to the following pointer for the driving scheme: the on time during the periodic driving voltage should be maintained long enough for the pixels to open to the maximum state of that voltage. One can also tune the on time to realize a different grey scale of the device. Our results also suggest that periodic and transient voltage pulses with the opposite polarity can be used to reset the trapped charge in the FP without a severe drop in the open ratio.

The transient regime becomes less and less noticeable as the frequency increases, as shown in Figure 7c. Based on the above discussion, this result implies that the asymmetric charge trapping and detrapping process plays a less significant role for these frequencies.

The continuous decrease of the open ratio, which appears to be similar to the backflow effect, reoccurs when the frequency increases to 50 kHz, 100 kHz, and 500 kHz, as shown in Figure 7c. One likely reason is that the high frequency of the driving voltage significantly impedes the relaxation of the trapped charge, leading to a continuously increasing degree of charge trapping. There may also be a limited transfer of ions through the water. It has been reported that water starts to behave as a dielectric when the frequency is above the critical frequency [6,35,36]. Equation (3) below is used to estimate the characteristic time constant for charge relaxation (*τ_C_*) in the configuration of a conductive drop confined between two conductive plates. *σ_l_*, *ε_l_* and *l* are the conductivity, the dielectric constant, and the height of the liquid, respectively; *σ_d_*, *ε_d_* and *d* are the conductivity, the dielectric constant, and the height of the dielectric, respectively [6].

(3) $$\tau_{C}=\varepsilon_{0}\frac{\varepsilon_{d}+\varepsilon_{l}\frac{d}{l}}{\sigma_{d}+\sigma_{l}\frac{d}{l}}\;\;\;\;\;\;$$

According to Equation (3), the estimated value for the critical frequency 1/*τ_C_* of the EWD device is on the order of magnitude of 1 kHz, given that *σ_l_* is on the order of 10^−6^ S/m, *σ_d_* is set to zero, *d* = 0.85 µm, *l* = 75 µm, *ε_d_* = 1.934, and *ε_l_* = 80. Hence, for the frequencies above 50 kHz, the transport of ions in the water and the subsequent charge accumulation at the water/FP interface is suppressed, affecting the electrowetting performance in the device severely.

**Figure 8:**
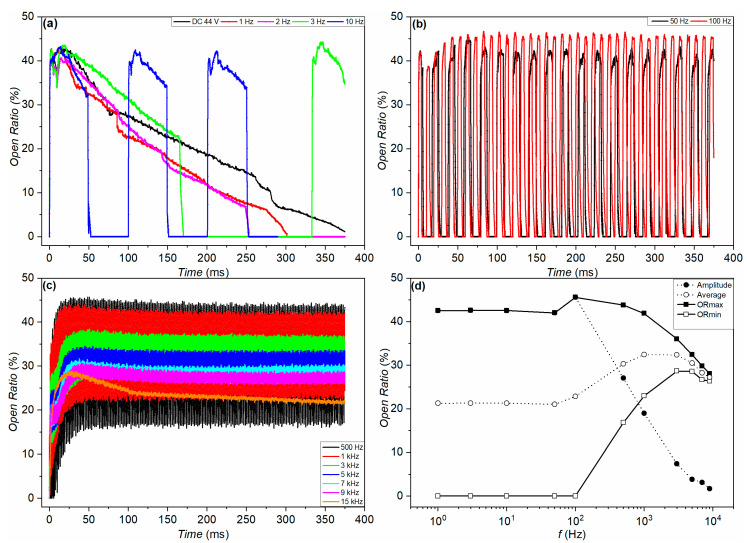
Open ratio response of the black-oil device. The device was driven by a square-wave voltage (0 V −> 44 V −> 0 V) of different frequencies. (**a**) Time dependence of the open ratio, measured at DC (black), 1 Hz (red), 2 Hz (magenta), 3 Hz (green), and 10 Hz (blue) (**b**) Time dependence of the open ratio, measured at 50 Hz (black) and 100 Hz (red). (**c**) Time dependence of the open ratio, measured at 500 Hz (black), 1 kHz (red), 3 kHz (green), 5 kHz (blue), 7 kHz (cyan), 9 kHz (magenta), and 15 kHz (orange). (**d**) Frequency dependence of the amplitude, average, maximum, and minimum of the open ratio.

The black-oil device generally shows similar frequency-dependent switching behavior to the purple-oil device, as demonstrated in Figure 8d. However, there is no noticeable transition regime for 50 and 100 Hz, as shown in Figure 8b, which is probably because the charge trapping and detrapping processes become less significant for the black-oil device when the driving frequency is lower than 100 Hz, compared to the purple-oil device. It has already been mentioned above that the electrical conductivity of the oil should not be neglected.

**Figure 9:**
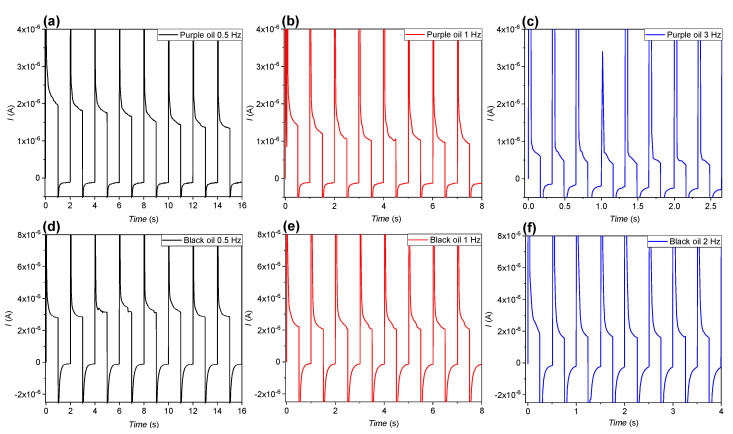
Current response of the purple- and black-oil devices. (**a**–**c**): The purple-oil device was driven by a square-wave voltage (0 V −> 25 V −> 0 V) of different frequencies. Current responses, measured at 0.5 Hz, 1 Hz, and 3 Hz, respectively. (**d**–**f**) The black-oil device was driven by a square-wave voltage (0 V −> 44 V −> 0 V) of different frequencies. Current responses, measured at 0.5 Hz, 1 Hz, and 2 Hz, respectively.

Figure 9a–c show the current response at 0.5, 1, and 3 Hz of the purple-oil device. Figure 9d–f show the current response at 0.5, 1, and 2 Hz of the black-oil device. When the voltage is applied (the first half-period), the current rises up to the maximum instantly and then decreases gradually. When the voltage is set to zero (the second half-period), the current instantly changes to a negative maximum and then approaches to zero gradually. The *I-t* curves show periodic charging and discharging behavior. In the first half-period, a relatively large current is present. The results of the purple-oil device distinctly show that the time constant of the second half-period is smaller than that of the first half-period. This additional timescale may be associated with charge trapping and detrapping, as charge trapping phenomena are known to be more strongly dependent on polarity. The black-oil device shows faster backflow (Figure 1c) compared to the purple-oil device (Figure 1a). As can be seen in Figure 8a, a timescale of backflow is typically 350 ms for the black-oil device. Since oil backflow is associated with the transport of charges through the oil phase, one expects to see this charge transport through the oil in the current characteristic (see Figure 9d–f). Indeed, a timescale of order 350 ms can also be found there. Interestingly, both the off- and on- period show a similar time constant. The similarity in the time constant can be understood by realizing that after the oil has backflowed completely, charges reside on the oil–dielectric interface, which are kept there by the applied voltage. If the voltage is switched, these charges are unloaded from the interface and will be transported through the oil film to the water phase. The current characteristic is typically that of a capacitor that is unloading, the *RC* time being governed by the resistivity of the oil, not unlike the oil backflow which is governed by the same resistivity.

The shape of the *I-t* curve does not change significantly with the period number. However, the current at the end of the first half-period decreases with the period number, which is noticeably shown in the results of the purple-oil device (Figure 9a–c) but not the black-oil device (Figure 9d–f). The decrease of the current of the purple-oil device probably results from a decrease of the leakage current when the device is open. In contrast, due to the fast oil backflow, the fast recovery of the oil film suppresses the leakage current of the black-oil device. The decrease of the leakage current is therefore weakened. Nevertheless, we are not clear why the leakage current of the purple-oil device decreases with the period number. It has been reported that there is a positive correlation between charge trapping in and the leakage current through dielectric materials [37,38,39,40,41].

The result in Figure 7a shows that there is already a decrease in the pixel closing speed (the slope of the open ratio during pixel closing) after 5 Hz, which suggests that the asymmetry between the charge trapping and relaxation already took place at the low frequency. We therefore suspect that the decrease of the current (see Figure 9a–c) is because the charges are trapped when the voltage is applied, but only part of them are relaxed when the voltage is zero, thereby continuously decreasing the number of charge trapping sites in the FP as the purple-oil device is opened and closed periodically. Simulation of the *I-t* property based on the equivalent circuit model shown in Figure 2 and the incorporation of an appropriate charge trapping model might be able to confirm this.

## 4. Conclusions

We fabricated the EWD devices with two types of oils and measured the current and open ratio responses to single and periodic voltage pulses. The measured open ratio responses to DC show that larger oil conductivity and negative voltage could lead to faster oil backflow. Rebound and reopening phenomena for negative driving voltages were also observed. A simplified RC model can explain the backflow as a result of a high oil conductivity, which is further confirmed by the contact angle measurements. Rebound and reopening phenomena are ascribed to the stronger adsorption (trapping) of the negative ions from the water in the FP. This may cause the observed faster backflow under application of a negative polarity. In AC driving, the oil backflow can be suppressed in a proper frequency range. We also observed a frequency-dependent transition regime where *OR*_min_ and *OR*_max_ increased gradually to reach a new stable switching stage, which probably resulted from the charge trapping effect.

The DC results suggest that the charge trapping and the charge leakage effects should be suppressed as much as possible. Hence, the oil phase should be minimally conducting. In addition, the dielectric material should show minimal charge trapping. Both the DC and AC results also suggest that an appropriate driving scheme may improve the stability of the optical response through a reverse of the deterioration in the open ratio. Applying transient voltage pulses with opposite polarity periodically can be used to reset the trapped charge in the FP. Since the charge trapping effect may influence the closing of the device, one may be able to find an appropriate resetting mode to achieve sufficient relaxation of the trapped charge without causing too large a drop in the open ratio. We would like to point out that, to fully understand the switching behaviors of the EWD devices, and to design a suitable driving scheme for the devices, it is necessary to carefully examine the charge trapping and charge conduction effects at the interfaces independently, and then incorporate the effects into the investigation of the electrowetting effect in the EWD devices. In the end, we believe that the experimental indications and discussions in this work provide meaningful results for other applications involving electrowetting behavior in liquid–liquid–solid systems, such as electrowetting retroreflectors [42] and an electrowetting lens [43].

## Supplementary Materials

The following are available online at https://www.mdpi.com/2072-666X/11/7/702/s1, Figure S1: Schematic diagram of the EWD device fabrication process; Figure S2: Purple dye formula; Figure S3: Cyan dye formula; Figure S4: Yellow dye formula; Figure S5: Magenta dye formula; Figure S6: Device and microscope images of the purple-oil device; Figure S7: Device and microscope images of the black-oil device; Figure S8: Schematic diagram of the EWOD sample fabrication process.
