# Supplementary material for: Oil Conductivity, Electric-Field-Induced Interfacial Charge Effects, and Their Influence on the Electro-Optical Response of Electrowetting Display Devices
Source: Micromachines (Basel). 2020 Jul 20;11(7):702. doi: 10.3390/mi11070702 (PMC7407153; doi:10.3390/mi11070702)
Supplement: Supplementary file 1 [file micromachines-11-00702-s001.pdf]

Supplementary Materials: Oil Conductivity, Electric-Field-Induced Interfacial Charge Effects, and Their Influence on the Electro-Optical Response of Electrowetting Display Devices

Chengdian Jiang, Biao Tang, Bojian Xu, Jan Groenewold and Guofu Zhou

EWD Device Fabrication

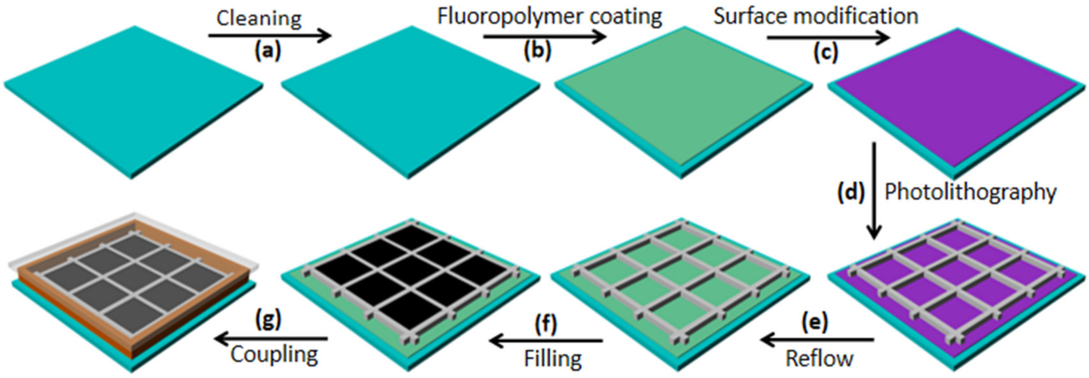

Figure S1. Schematic diagram of the EWD device fabrication process. (**a**) The ITO/glass substrate was cleaned in the standard cleaning line. (**b**) A layer of Fluoropolymer (FP) was spin-coated on the substrate. (**c**) The FP surface was activated using reactive ion etching (RIE) to change the hydrophobic state to the hydrophilic state. (**d**) A layer of n-type photoresist (PR) was then spin-coated on FP. After the photolithography process, the pixel walls were formed. (**e**) The substrate was heated to reverse the FP surface back to the hydrophobic state. (**f**) The colored oil was then uniformly filled into the pixels through a raster filling method under water. (**g**) The cells were filled with water and coupled with a top ITO/glass plate.

Dyes Information

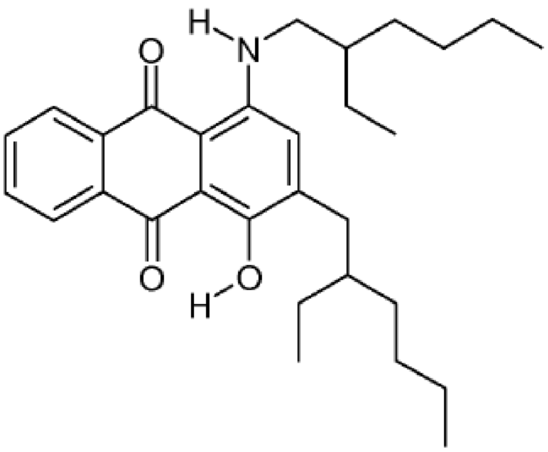

Figure S2. Purple dye formula.

Molecular weight of the purple dye is 463, the synthesis process is outlined below: 50 g 1,4-dihydroxyanthraquinone, 1.0 g sodium dodecyl sulfate, 1 L water were mixed in a 2 L reaction flask. Subsequently, 50 g Na_2CO_3 and 50 g $Na_2S_2O_4$ was being added to the flask. The solution was heated

to 80 °C and reacted for 0.5 h. When the solution completely turned to yellow, TLC was used to track the progress of the reaction (n-hexane: acetone = 3, Rf = 0.68). If the reaction was not completed, more alkali and $Na_2S_2O_4$ must be added to the solution. After that, the solution was filtered and dried at vacuum (60 °C) to obtain intermediate C'-1 with the yield of 95%. 50 mL isopropanol, 24.2 g (242 g/mol, 0.1 mol) C'-1, 32.0 g (128 g/mol, 0.25 mol) 2-ethylhexanal and 4.35 g (145 g/mol, 0.03 mol) phospholipid acetate were mixed in a three-necked flask with agitation, and the solution was then heated to 80 °C under an N_2 atmosphere. After reacting for 20 h, the solution was cooled to −20 °C. Then 200 mL industrial ethanol (95%) was poured into the solution, stirred and then cooled for 3 h. The second intermediate C'-2 was obtained with 88.6% yield. 50 mL ethanol (95%), 5.28 g (352 g/mol, 0.015 mol) C'-2, 9.5 g (129 g/mol, 0.073 mol) 2-ethylhexylamine and 1 mL concentrated hydrochloric acid were mixed in a three-necked flask with agitation. Then 1.95 g (65 g/mol, 0.03 mol) zinc powder was added to the solution under an N_2 atmosphere, the solution was heated to 60 °C and reacted for 2 h. After that, 10 mL of KOH solution (1.6 g, 56 g/mol, 0.03 mol) was slowly poured into the above solution, and the solution was oxidized for 30 min. Solid impurities was removed by filtration, solvent was removed by rotary evaporation, and the product P-1 was purified by column chromatography.

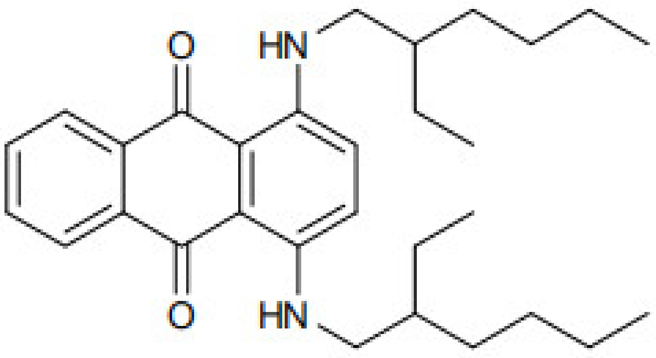

Figure S3. Cyan dye formula.

Molecular weight of the cyan dye is 462.

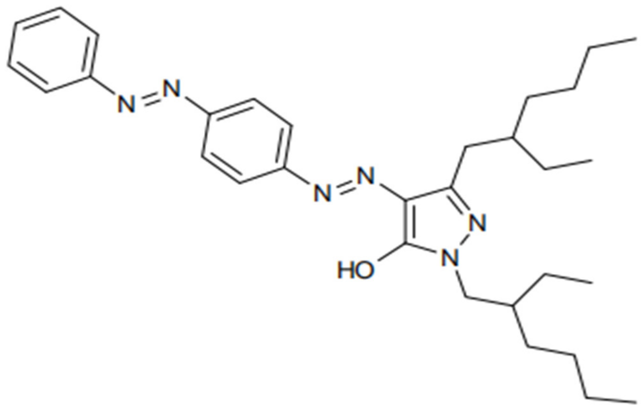

Figure S4. Yellow dye formula.

Molecular weight of the yellow dye is 516.

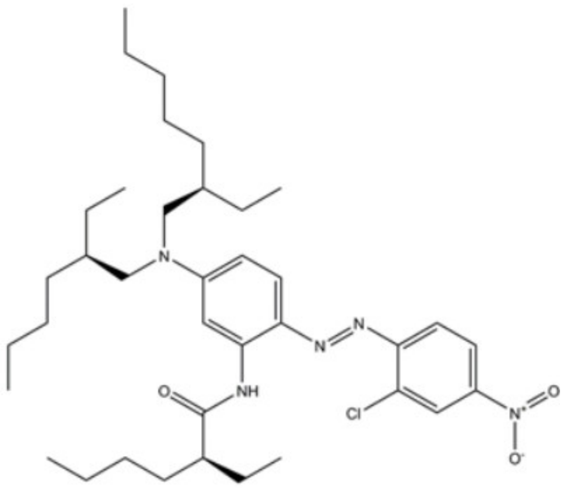

Figure S5. Magenta dye formula.

Molecular weight of the magenta dye is 655.5, with the synthesis process as below: 3.16 g (158 g/mol, 0.02 mol) $C_8H_{10}N_2O$, 11.5 g (192 g/mol, 0.06 mol) $C_8H_{17}Br$ and 5.5 g (138 g/mol, 0.04 mol) K_2CO_3 were mixed in 70 mL DMAc. The solution was heated to 140 °C under an N_2 atmosphere. After a reaction for 9 h, the solution was cooled to room temperature, and poured into 100 mL water to precipitate oily liquid. After that, the solution was extracted with $C_4H_8O_2$, concentrated and purified by column chromatography to obtain the intermediate with the yield of 35%. 7.48 g (374 mol/L, 0.2 mol) intermediate was mixed in 200 mL industrial ethanol. 16 mL (12 mol/L, 0.2 mol) concentrated hydrochloric acid was dissolved in the solution, then the solution was heated to 80 °C and reacted for 2 h under an N_2 atmosphere. TLC ($C_4H_8O_2/C_5H_{12}$ = 1/5) was used to detect the progress of the reaction. When the reaction was completed, NaOH solution was added to neutralize the acidity in solution. Then the solution was extracted by petroleum ether, washed twice and concentrated to remove solvent, the hydrolyzate with the yield of 95% was obtained. 6.6 g (332 g/mol, 0.02 mol) hydrolyzate and 4 g (101 g/mol, 0.04 mol) triethylamine were mixed in 200 mL DMF. 3.2 g (162 g/mol, 0.02 mol) lsooctyl chloride was slowly added into the solution. After reacting for 1 h, the solution was extracted by petroleum ether and washed twice. The intermediate was obtained with a yield of 95%. 1.72 g (172 g/mol, 0.01 mol) O-chloro-p-nitroaniline was mixed in 10 mL concentrated phosphoric acid. 4.0 g (0.013 mol, 40%) nitrosyl sulfate was added in the solution. After the solution was diazotizated of 2 h. 4.58 g (458 g/mol, 0.01 mol) intermediate was mixed in 50 mL industrial ethanol. The solution was cooled to 0–5 °C and coupled for 2 h. After that, the solution was poured in 100 mL water, ethyl acetate was used to extract the Magenta dye. The solution was purified by column chromatography, with a purification yield of 60%.

Device and Microscope Images

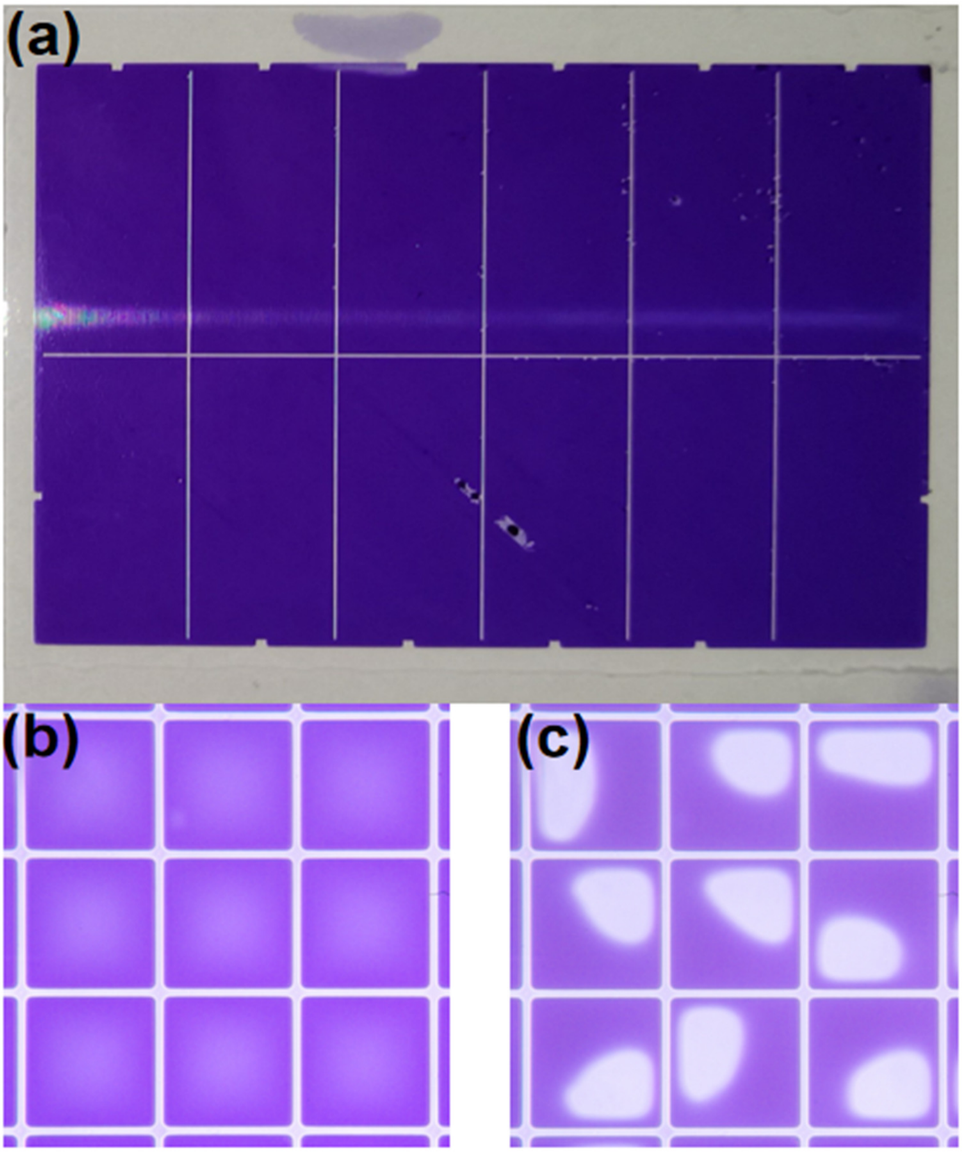

Figure S6. (**a**) Photo of the purple-oil device. (**b**) Microscopic image showing the close state of the purple-oil device. (**c**) Microscopic image showing the open state of the purple-oil device.

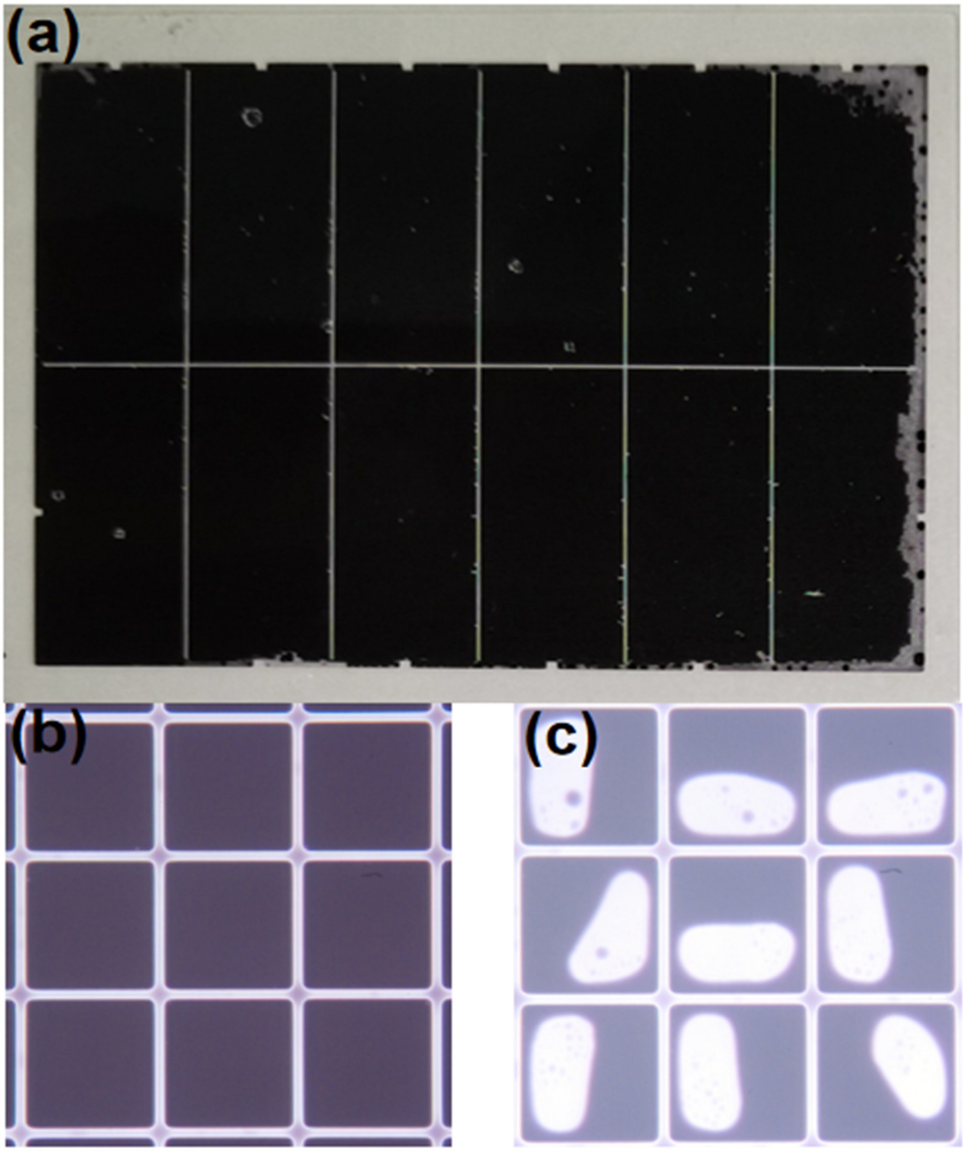

Figure S7. (**a**) Photo of the black-oil device. (**b**) Microscopic image showing the close state of the black-oil device. (**c**) Microscopic image showing the open state of the black-oil device.

EWOD Sample (for Contact Angle Measurement) Fabrication:

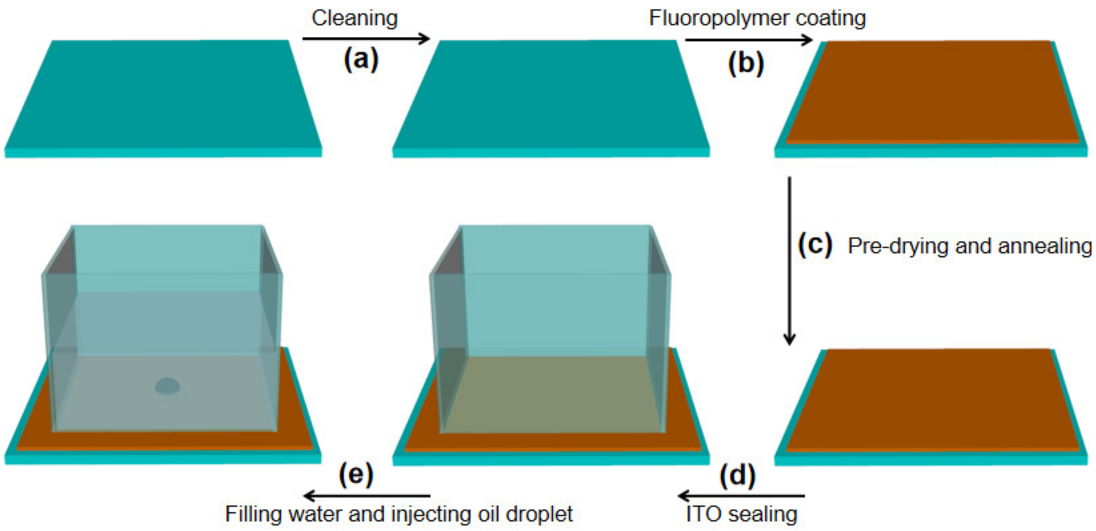

Figure S8. Schematic diagram of the EWOD sample fabrication process. (**a**) The ITO/glass substrate was cleaned in the standard cleaning line. (**b**) A layer of Fluoropolymer (FP) was spin-coated (1000 RPM for 60 s) on the substrate. (**c**) The FP layer was pre-dried on hot plate (85 °C) for 3 min, then was annealed in an oven (185 °C) for 30 min. (**d**) The surface of the insulating layer was sealed to form a container. (**e**) Water was filled into the container, and then oil droplet was injected onto the FP substrate.
